# Medical Flight Emergencies and Bias: #thatisbias #whatadoctorlookslike #ILookLikeaDoctor

**DOI:** 10.1089/heq.2020.0006

**Published:** 2020-06-16

**Authors:** Comilla Sasson, Tamika K. Cross, Fatima Cody Stanford

**Affiliations:** ^1^ Department of Emergency Medicine, University of Colorado, Aurora, Colorado, USA.; ^2^ Department of Health Services Research, Colorado School of Public Health, Aurora, Colorado, USA.; ^3^ Department of Obstetrics and Gynecology, University of Texas-Houston, Texas, USA.; ^4^ Division of Endocrinology-Neuroendocrine, Department of Medicine, MGH Weight Center, Massachusetts General Hospital, Boston, Massachusetts, USA.; ^5^ Department of Pediatric-Endocrinology, MGH Weight Center, Massachusetts General Hospital, Boston, Massachusetts, USA.; ^6^ Nutrition Obesity Research Center at Harvard (NORCH), Harvard Medical School, Boston, Massachusetts, USA.

**Keywords:** bias, discrimination, race, medical emergencies

## Abstract

One in 604 flights will have a medical emergency. With 87,000 flights per day in the United States alone, that is ∼144 medical emergencies per day. When a passenger has a medical emergency in-flight, do staff respond with equity to persons who offer assistance? Unfortunately, several news stories have highlighted race and gender bias against woman physicians of color who come to the aid of a person in distress while in-flight. Three separate stories have ignited a national conversation about what it means to “look like a doctor.” In this article, we profile three vignettes of women physicians of non-white race that challenges the notion that all doctors are treated equally when trying to assist passengers who are experiencing a medical in-flight emergency. We share stories of how bias has affected other health care providers in similar situations. Some physicians have not been asked anything but their name, whereas others are questioned for their credentials before they can assist. In other vignettes, even with valid credentials, these offers of assistance from physicians are rebuked. We will challenge the aviation industry to put passengers first by training flight crews to see and address implicit and explicit biases, standardize protocols to remove barriers for assistance, challenging the notion of paperwork superseding care, and changing a very broken process that is inconsistent at best.

Is there a doctor on-board? One in 604 flights will have a medical emergency.^1^ With 87,000 flights per day in the United States, that is ∼144 medical emergencies per day. Most commonly in-flight medical emergency complaints include syncope (33%), gastrointestinal (15%), respiratory (10%), and cardiovascular (7%) complaints.^2^ Although the duty to respond by health care professionals varies by country, medical providers are covered in the United States by the Aviation Medical Assistance Act,^3^ which provides Good Samaritan coverage for the health care provider, unless there is gross negligence.

Unfortunately, there have been several stories that have highlighted the bias that exists when a person of color or a woman comes to the aid of a person in distress while in-flight. In the era of social media, some of these stories receive not only local attention, but some stories have also reached a level of national and international prominence.^4–6^ They evoke a conversation about the role of racism and gender inequality when physicians who represent these demographics volunteer to offer their assistance. We highlight three stories of women physicians of color who attempted to assist passengers during in-flight medical emergencies.

In 2016, on a flight from Detroit to Houston, T.K.C., an obstetrics-gynecology resident physician, raised her hand to help a passenger who was unresponsive. She was immediately met with resistance. The Delta flight attendant said, “Oh wow, you're an actual physician? Let me see your credentials. What type of doctor are you? Where do you work? Why were you in Detroit?”

The flight attendant would not allow her to assist. As a resident physician, she did not have a medical license to prove her medical degree. Later, a white male passenger who stated he was a physician approached the flight attendant. The flight attendant turned to T.K.C. and said, “Thanks for your help, but he can help us, and he has his credentials.” The man never showed any credentials.

T.K.C. shared her experience of racial bias on social media, igniting the hashtag #whatadoctorlookslike to challenge the stereotypes many women of color face. She also spent 2 months after the event convincing Delta executives to change their policies. Delta announced, effective December 1, 2016, the company would no longer require verification of medical credentials from health care professionals helping on-board medical flight emergencies and pledged to train its frontline employees on “inclusion training.” T.K.C.'s experience spurred a national conversation and served as a launchpad for policy surrounding her struggle to deliver timely care to a person mid-flight.

In October 2018, the Federal Aviation Administration (FAA) Reauthorization Act of 2018 was passed by Congress. In Section 407, it requires “The Comptroller General to report to Congress within the next six months on each air carrier's training policy for its employees and contractors regarding racial, ethnic, and religious nondiscrimination.”^7^ The section requires the Secretary of Transportation to “develop and disseminate to air carriers best practices necessary to improve”^7^ nondiscrimination training secondary to recent events that cast a doubt upon equity in treatment of passengers on commercial airline carriers.

Immediately after the passage of the FAA Reauthorization Act of 2018 at the end of October in 2018, F.C.S. was on a Delta flight from Indianapolis to Boston when a woman required medical assistance in-flight. F.C.S. began helping the patient seated next to her. One flight attendant asked whether she was a physician. She replied yes, and immediately produced her medical license from her wallet. As she continued to assist the patient, a second flight attendant approached and once again asked whether she was a physician. Just as she had done before, she produced her medical license. After a few minutes, she was approached by both flight attendants. The first flight attendant asked “Are you a head doctor? Confused by what a “head doctor” was, she stated “I am unsure what you mean by “head doctor.” Then the second flight attendant immediately asked, “Are you actually an M.D.? Is this your license?” She replied to the flight attendant saying, “Why would I carry someone else's medical license?”

Like T.K.C., F.C.S. took to social media to share her story and showcase the in-flight racism she experienced trying to help a passenger in need. Delta replied saying this was a partner airline, and they would make sure employees were aware of their policy that did not require health care professionals to show proof of credentials.

Just 6 weeks after F.C.S.'s incident, C.S. was on a United Airlines flight from Denver to Houston. She heard someone fall to the ground and immediately rushed to help. Remembering the experiences of T.K.C. and F.C.S., she was careful to announce she was an emergency medicine physician three separate times to the flight attendants. The flight attendant would not allow her to assist the patient lying on the ground in the aisle until she showed her medical credentials. C.S. proceeded to care for the patient, despite the objections of the flight attendant. After the patient was escorted off the flight by paramedics, the flight attendant came to C.S.'s seat to obtain her credentials. The flight attendant said to C.S., “[You] acted like you knew what you were doing, so I thought you probably were a doctor, so it was ok with me to have you show your credentials after.”

C.S. shared her story with her local news outlet and social media, reigniting a national conversation about the biases women physicians of color face when helping passengers in-flight.

## Implicit Versus Explicit Bias

Explicit bias is defined as “when a person is aware of his/her evaluation of a group, believes that evaluation to be correct in some manner, and has the time and motivation to act on it in the current situation.”^8^ It is generally deemed socially unacceptable and is not tolerated in most situations. However, implicit bias is more unconscious and subtle. “[It] does not require the perceiver to endorse it or devote attention to its expression…bias can be activated quickly and unknowingly by situational cues (e.g., a person's skin color or accent), silently exerting its influence on perception, memory, and behavior.”^8^ Are the aforementioned vignettes implicit versus explicit bias? It is often difficult to tell; however, there are ways in which both can be addressed by health care professionals and the aviation industry.

The FAA Reauthorization Act of 2018 is a first step in acknowledging the racial/ethnic, and gender discrimination that was apparent in the interactions of these three female physicians. National organizations and congressional leaders have drawn attention to the discrimination passengers experience on flights. However, more should be done to address explicit and implicit biases, which affect the quality of care (timeliness of response, delayed intervention, etc.) passengers receive while in-flight (Table 1).

**Table 1.** Health Care Professional and Aviation Industry Potential Strategies to Address Bias in In-Flight Emergencies

**What health care professionals can do:**
*Speak up.* When injustices happen in-flight, use social media, news outlets, and organized professional medical organizations to speak out about the situation. Change cannot happen without first raising awareness of the situation.
*Help if help is needed.* Put the needs of the patient first and foremost. If you are able to assist, do so first, as time can be critical in caring for a sick person.
*Carry your medical license or credentials.* This is a secondary measure. Carry proof of your credentials in your wallet or in your smartphone.
*Engage in conversations with your colleagues about implicit and explicit bias.* Everyone has biases. If we do not address these openly and honestly whenever we encounter these biases, whether it is at the grocery store or in the airplane, we will never counter these attitudes and behaviors.
*When you or someone else experiences bias, call it what it is.* Discrimination and bias continue to exist because we are afraid to be controversial or stand up for ourselves. Stand up for others when you see someone engaging in a discriminatory practice.
*Recognize and change your own biases.* Whether they are implicit or explicit, take the time to explore what drives your own perceptions of stereotypes, judgments, and biases when interacting with others. Come up with a plan on how to overcome these biases.
*Serve as an ally.* If you are a person from a more privileged group (i.e. white male), speak up against injustices. Persons from these groups have a powerful voice to help drive change.
**What the aviation industry can do:**
*Move beyond cursory “inclusion and diversity training.”* Addressing people's implicit and explicit biases will not be corrected in a brief training. This commitment to change has to be an ongoing discussion and dialogue to change the pervasiveness of the discrimination that occurs daily.
*Train flight crews to see and address implicit and explicit biases.* While in-flight, flight attendants are entrusted with providing service and assisting passengers to have a carefree aviation experience. They can use the power they have been granted to address and recognize bias and ensure in-flight medical emergencies are handled in the best possible manner.
*Develop and implement plans to address discriminatory practices from flight crews.* Recognition and awareness are the first steps for change. Understanding how these situations occur and equipping teams with plans to prevent this from arising are key.
*Data analysis and appropriate feedback to flight crew members.* When situations arise, track these data, provide appropriate and timely response to passenger concerns.
*Standardize protocols to remove barriers for assistance.* Ensure all flight crews are up-to-date on how an in-flight medical emergency should be handled.
*Accept verbal confirmation of profession and willingness to assist.* Medical professionals, unless committing gross negligence, are covered by Good Samaritan laws. Allow them to assist the patient first; complete the paperwork second.
*Preregister medical professionals at the time of buying their tickets if they are inclined to help in the case of an in-flight medical emergency.* This voluntary measure may help alleviate questions regarding one's credentials.

Commercial aviation is one of the safest modes of transportation because it has used data, training, advanced technology, regulations, and closed-loop communication to make airplanes safe. We challenge the aviation industry to (1) make passengers feel safe, valued, and equally important; (2) not tolerate instances of discrimination; and (3) let physicians do our job and care for patients in need.

## Abbreviation Used

FAA: Federal Aviation Administration
